# Exploring the Relationship Between Adipose Tissue and Alzheimer’s Disease Pathogenesis

**DOI:** 10.1002/alz.092413

**Published:** 2025-01-03

**Authors:** Miriam Bettinetti‐Luque, Laura Trujillo‐Estrada, Juana Andreo‐Lopez, Cynthia Campos‐Moreno, Ivan Fatuarte‐Juli, Mario Morales‐Cabello, Celia Da Cunha, Elisabeth Sanchez‐Mejias, Frank M LaFerla, Antonia Gutierrez, David Baglietto‐Vargas

**Affiliations:** ^1^ University of Malaga/CIBERNED/IBIMA, Málaga Spain; ^2^ University of California, Irvine, Irvine, CA USA

## Abstract

**Background:**

Alzheimer’s disease (AD) is a complex disorder and multiple cellular and molecular mechanisms are involved in AD onset and progression. Recent evidences have suggested that metabolic alterations are an important pathological feature in disease progression in AD. Likewise, diabetes and obesity, two mayor metabolic illnesses, are risk factors for AD. These two overwhelming diseases are associated with a significant expansion of visceral adipose tissue. Here, we hypothesize that the visceral adipose tissue may serve as a key communicator organ between the brain and peripheral metabolic illnesses and affecting both types of disorders.

**Method:**

We used histological stains, immunohistochemistry and biochemical means to determine changes in the visceral adipose tissue from WT and db/db mice. Moreover, similar techniques were used in 3xTg‐AD mice that received white fat pads from WT and db/db donors to determine any changes in amyloid and tau pathology.

**Result:**

Our study shows that recipient 3xTg‐AD mice from db/db fat pads mice develop profound changes in tau pathology due to increased CDK5 expression compared to 3xTg‐AD mice that received fad pads from WT mice. This increment in tau level was associated with elevated levels in IL‐1β and profound microglia activation. Moreover, we found the opposite effect on amyloid pathology, in which insoluble Aβ levels and Thioflavin positive plaques were reduced in recipient 3xTg‐AD mice from db/db fat pads compared to 3xTg‐AD mice that received fad pads from WT mice. These reduction in Aβ levels were correlated with an increment in microglia phagocytic capacity.

**Conclusion:**

Overall, our study demonstrate a novel important crosstalk between Alzheimer´s disease and obesity/diabetes type II through visceral adipose cells and a differential effect on tau and Aβ pathology mediated by an activated immune response.

This study was supported by Minister of Science and Innovation grant PID2019‐108911RA‐100 (D.B.V.), Alzheimer’s Association grant AARG‐22‐9282/9, Beatriz Galindo program BAGAL18/00052 (D.B.V.), University of Malaga grant PPIT.UMA.B1‐2021_32(LTE) and Institute of Health Carlos III (ISCiii) grant PI18/01557 (A.G.) co‐financed by FEDER funds from European Union.

